# Structural insights into human angiogenin variants implicated in Parkinson’s disease and Amyotrophic Lateral Sclerosis

**DOI:** 10.1038/srep41996

**Published:** 2017-02-08

**Authors:** William J. Bradshaw, Saima Rehman, Tram T. K. Pham, Nethaji Thiyagarajan, Rebecca L. Lee, Vasanta Subramanian, K. Ravi Acharya

**Affiliations:** 1 Department of Biology and Biochemistry, University of Bath, Claverton Down, Bath BA2 7AY, United Kingdom

## Abstract

Mutations in Angiogenin (*ANG*), a member of the Ribonuclease A superfamily (also known as RNase 5) are known to be associated with Amyotrophic Lateral Sclerosis (ALS, motor neurone disease) (sporadic and familial) and Parkinson’s Disease (PD). In our previous studies we have shown that ANG is expressed in neurons during neuro-ectodermal differentiation, and that it has both neurotrophic and neuroprotective functions. In addition, in an extensive study on selective ANG-ALS variants we correlated the structural changes to the effects on neuronal survival and the ability to induce stress granules in neuronal cell lines. Furthermore, we have established that ANG-ALS variants which affect the structure of the catalytic site and either decrease or increase the RNase activity affect neuronal survival. Neuronal cell lines expressing the ANG-ALS variants also lack the ability to form stress granules. Here, we report a detailed experimental structural study on eleven new ANG-PD/ALS variants which will have implications in understanding the molecular basis underlying their role in PD and ALS.

Angiogenin (ANG), a 14-kDa basic protein is an angiogenic ribonuclease and member of the bovine pancreatic ribonuclease (RNase A) family (also known as RNase 5)^1^,^2^,^3^. Like RNase A, ANG cleaves preferentially on the 3′ side of pyrimidines and follows a transphosphorylation/hydrolysis mechanism^4^,^5^. In addition, the crystal structure of human ANG has revealed that it has an RNase A fold and the catalytic triad (residues H13, K40 and H114) is conserved (Fig. 1)^6^,^7^,^8^. The enzymatic activity of ANG is several orders of magnitude lower than that of RNase A towards conventional RNase substrates^5^, but essential for its angiogenic effect^3^,^9^.

The mechanism of ANG action is not yet fully understood, but appears to involve different pathways, including receptor binding on endothelial cells^10^, nuclear transport^11^,^12^ and possible activation of proteolytic enzymes and cascades^13^. It is now established that *ANG* plays important roles in various physiological and pathological processes. For a recent comprehensive review on ANG, see Sheng and Xu^14^. In a recent report it has also been shown that ANG can promote hematopoietic regeneration by dichotomously regulating quiescence of stem and progenitor cells^15^.

Apart from ANG’s role in neovascularization, *ANG* has been shown to be a neurotropic and neuroprotective factor^16^,^17^ and ANG variants have been implicated in ALS and Parkinson’s disease. Greenway *et al*.^18^,^19^ reported that mutations in *ANG* in ALS, both familial and sporadic. Since these first reports, several other studies worldwide have also identified *ANG* mutations in ALS patients^20^,^21^,^22^,^23^,^24^,^25^,^26^,^27^,^28^,^29^. Furthermore, a large multi-site study reported the association of mutations in ANG with ALS as well as Parkinson’s disease (PD)^21^,^30^,^31^ (Table 1). In parallel, ANG has also been shown to have a neuroprotective effect in a mouse model of PD^32^,^33^.

While human ANG is also shown to be upregulated especially in prostate cancers and glioblastomas^34^, it is downregulated in ALS and PD^35^. At the cellular level, it has also been established that, in response to growth stimuli, ANG undergoes nuclear translocation and stimulates ribosomal RNA (rRNA) transcription, which is essential for cell growth and proliferation^36^. Further findings established that ANG is a stress-activated RNase that cleaves tRNA fragments which can inhibit translation initiation and is a key to stress response and cell survival^37^,^38^,^39^,^40^. Thus, it is quite likely that excessive ANG action underlies uncontrolled cell proliferation and sustained growth as manifested in cancer whereas its insufficiency might be a pathogenesis of degenerative diseases which are characterized as decreased survival and premature death^35^.

In order to elucidate the role of ANG mutations in ALS, we have shown previously that some ANG variants have significant reduction in RNase activity which is correlated with a reduction in cell proliferation and angiogenic activities^41^. These results formed the basis for understanding the role of ANG in ALS and neuronal differentiation and a definitive role for ANG in neurite growth and pathfinding was established^16^. We have also shown that ANG is a neuroprotective factor and ALS-associated ANG variants affect neurite extension/pathfinding and survival of motor neurons^17^. In a more recent comprehensive study we have provided (for the first time) detailed structural and molecular insights into the mechanism of action of eleven human ANG-ALS variants in neurons^8^. In this report, we have extended our structural study to eleven new human ANG PD/ALS variants and provide the detailed molecular features of these variants in comparison with the normal ANG protein, thus progressing from understanding structure to using structure to understanding disease.

## Results and Discussion

We have determined the crystal structure of eleven ANG-PD/ALS variants H13R, K40R, K54R, K60E, Q77P, T80S, R95Q, F100I, V103I, H114R, R121C (Table 2, in the resolution range 2.3–1.35 Å) and elucidated the structural changes in the key functional regions of the variants by comparing them with the high resolution structure of native (wild-type) ANG (reported previously by us at 1.04 Å)^8^, (Tables 3 and 4). We have also assessed the catalytic (RNase) activity (i.e., activity toward tRNA) of ANG PD/ALS variants which showed diminished level of RNase activity, in the range of 0.0–80% compared with the native ANG protein except variant R121C which showed enhanced activity of 131% (Table 4).

As reported previously, the active site of ANG consists of several sub-sites (as originally described for RNase A): a P_1_ substrate binding site involved in phosphodiester bond cleavage; a B_1_ site for binding the pyrimidine whose ribose donates its 3′ oxygen to the scissile bond; a B_2_ site that preferentially binds a purine ring on the opposite side of the scissile bond; and additional sites for peripheral bases and phosphates^6^.

ANG residues H13, K40 and H114 (part of the P_1_ sub-site) correspond to the catalytic triad. Replacements of H13 or H114 by Ala^3^ or K40 by Gln^9^ decrease ribonucleolytic activity by factors of at least 10^4^, 10^4^ and 2 × 10^3^, respectively. These early findings established that the roles of the three ANG residues are similar to those of their counterparts in RNase A.

In the crystal structure of H13R ANG PD variant, the substitution of H13 by Arg is very clear in the electron density map and the overall structure is largely the same as with native ANG. In the native structure, N^δ1^ of H13 forms a hydrogen bond with the main-chain O of T44 and N^ε2^ makes a water-mediated interaction with the amide nitrogen atom of L115 (Table 3). In the variant, the Arg side chain stretches out into the active site pocket making a direct interaction with Q117 while retaining its interactions with residues T44 and L115 (Fig. 2). The interaction with Q117 (known to be the obstructive pyrimidine-binding site residue in ANG)^6^,^42^ seem to result with slight rearrangement of the C-terminal helix. In addition, in the variant, the Arg side chain is not optimally positioned to interact with the two other catalytically important residues K40 and H114. This provides a structural explanation for the dramatic loss of RNase activity observed in this variant.

K40 is a key residue at the catalytic centre of ANG and adopts multiple conformation in the native structure. However the conservative substitution of Arg for K40 reduces the activity to ~6%, similar to the effect of the K41 to Arg mutation in RNase A^43^. In the crystal structure of the ALS variant K40R, the Arg side chain possesses a single conformation and several key interactions of K40 (with residues Q12, C39, N43 and T44, which are integral to the catalytic site) known to be important for the stabilization of the pentavalent transition state during RNA cleavage are lost (Table 4, Fig. 2). Instead, in the variant, the Arg side-chain makes direct interaction with Q112 by reorienting its side chain (more solvent exposed, Table 4).

In the H114R ALS variant the protein retains only 2% of the enzymatic activity toward tRNA. H114 is part of the catalytic site (P_1_) in the native structure and makes direct interaction with D116. However, in the variant structure (Fig. 2) this interaction is lost and a new van der Waals interaction with L69 has been observed involving the flexible loop region H65-R70. The main chain of R114 also makes an interaction with Q12 (which is known to be part of the substrate-binding site in the native protein). The loss of interaction with D116 also results with movement of C-terminal residues beyond D116 thus causing destablization in the variant structure (where the Arg side chain is more solvent exposed compared with the His residue in the native structure, Table 4) which will have major impact on the loss of catalytic activity as these residues are implicated in substrate recognition.

K54 does not participate or belong to any of the known catalytic sub-sites of ANG^6^. The variant K54R retains ~49% of enzyme activity (Table 4). In this PD variant structure this residue is exposed on the surface of the molecule. As described previously in the case of K54E ALS variant structure^8^, it is likely that the basic nature of the mutated residue in the variant will have an influence on the catalytic activity through a new interaction with residue Y6 (Fig. 2) and a slight rearrangement of the N-terminal helix involving the catalytically important residue H13. Also, in the variant structure the Arg side chain makes a water mediated interaction with R51.

**Figure 1:**
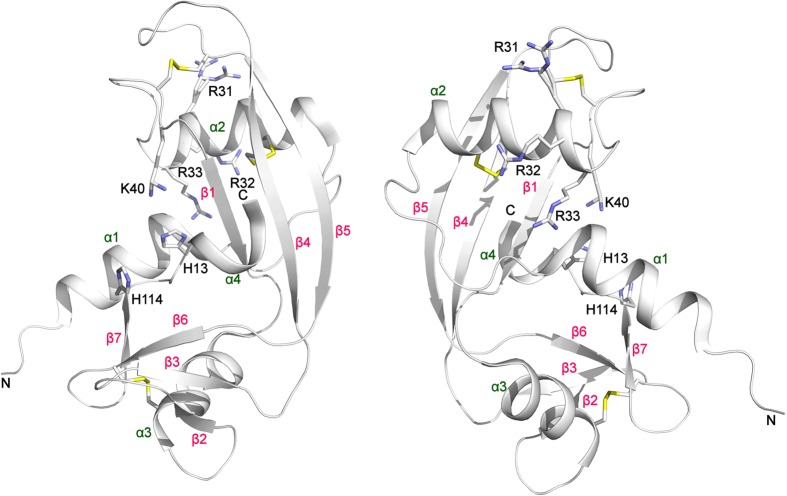
Structure of ANG with ALS/PD variants marked. The active site residues (H13, K40, H114) and the nuclear localization sequence (R31, R32, R33) are shown and are labelled in black along with the N- and C-termini. The disulphide bridges are also shown. K40 and R31 both show multiple conformations. Helices are labelled in dark green and strands in pink. Figures were created using the program PyMOL (http://www.delanoscientific.com)

**Figure 2:**
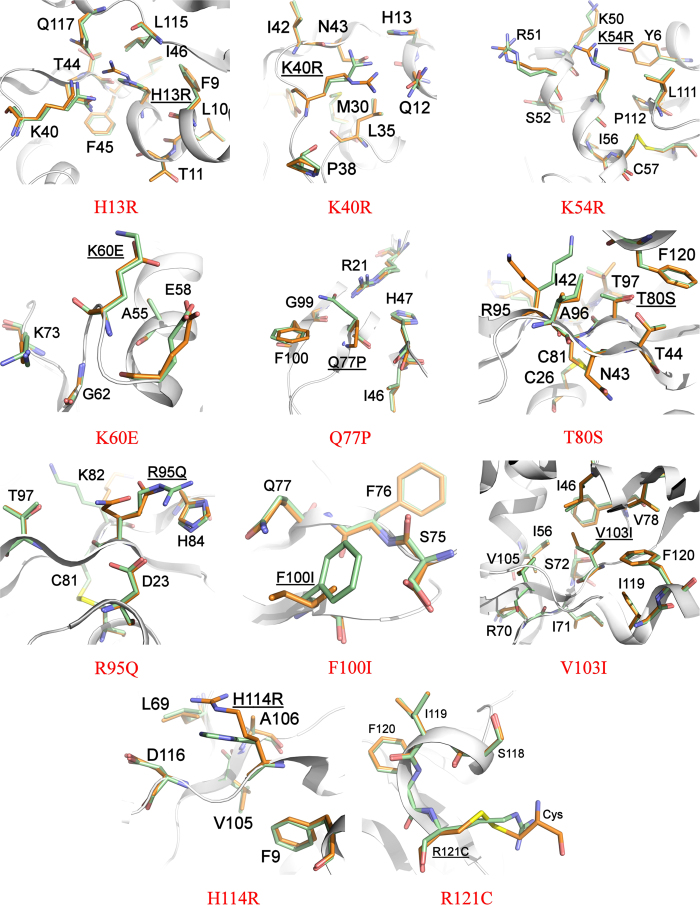
Structural comparison of ANG and ALS/PD-associated variants. Superposition of native ANG (in green) and ANG-ALS/PD variants (in orange). Residues that exhibit hydrogen bonding or Van der Waals contacts with the ANG-ALS/PD residues in either native ANG or mutant structures are also shown. The variant residues are underlined. The cysteine from the glutathione bound to R121C variant is also shown.

ANG residues 60–68 are part of an external region that includes strand B2 and loops on either side (Fig. 1)^6^. Based on known experimental data to date this region has been predicted to form part of the cell receptor binding site^44^. K60 forms part of this region at one end of the segment and is located on the surface of the molecule in the 3D structure. Mutation of the residue to Glu retains ~80% of the enzymatic activity. Previously it has been shown that chemical modification of K60 retained 34% activity^9^ and this surface residue has minor catalytic role. In the K60E PD variant structure the mutated residue gains a new interaction with E58 (Fig. 2), which seems to result in a level of repulsion of neighbouring residues in comparison to the wild-type ANG structure.

The residue Q77 forms part of the β4 strand in the ANG wild-type structure. So far there is no assigned role for this residue. However, in the Q77P PD variant structure (which contains 4 molecules in the asymmetric unit with clear electron density for the proline ring, which is less accessible to solvent) the hydrogen-bond (2.9 Å) observed between Q77 and R21 (part of helix α2) is lost (Fig. 2) thus creating a hydrophobic environment. However, in the variant structure, residue P77 also retains van der Waals contacts with residues I46 and F100 that are in close proximity to the substrate binding site (Table 3). However, these structural changes might contribute for the loss (~40%) of enzyme activity (Table 4).

In the native ANG structure, a hydrogen bond links the O^γ1^ atoms of T44 and T80^6^. In addition, based on mutagenesis and structural studies it was proposed previously that the strong cytidine preference of T80A is attributable to the intrinsic ability of T44 to form a much more effective hydrogen bond with N3 of cytidine than with that of uridine nucleotides^45^,^46^. Thus in native ANG, the T44-T80 hydrogen bond selectively weakens the interaction between T44 and N3 of cytosine. In the T80S ALS variant structure, the position of S80 is well defined in the electron density map and its atoms occupy positions essentially the same as in those of the corresponding atoms in native ANG. In particular, the position of O^γ1^ atom of T80 overlaps with the equivalent position of O^γ^ atom of S80 in the variant structure (more solvent accessible, Table 4) and retains most of the interactions seen in the wild-type structure. However, a significant number of van der Waals contacts surrounding residue T44 are lost in the variant structure. In addition, the absence of C^γ2^ atom in the variant structure meant that most of the van der Waals hydrophobic interactions with I42 are lost as well. It is quite likely that this results in slight reduction in stability, causing the observed loss in catalytic activity (~22%, Table 4, Fig. 2).

R95 is located on the surface of the molecule in the structure of ANG. It forms part of the β5 strand and interacts with K82 and H84 from the neighboring β4 strand^6^. Based on previous mutagenesis studies, both K82 and H84 have been shown to have minor roles in catalysis. In the PD variant structure R95Q (Fig. 2), the Gln side chain is flexible (can be observed in two possible conformations in the electron density map). However, shortening of the side chain length in the variant seems to have resulted with loss of interactions with H84 thus providing a possible explanation for the diminished catalytic activity of 52% (Table 4).

In the native ANG structure F100 and V103 residues (located on β5 and β6 strands respectively, Fig. 1) are in close proximity to the substrate binding site involving residue E108. The results from two variant structures F100I and V103I are analyzed here and both the variant residues are well defined in their respective electron density maps. The F100I variant implicated in ALS has retained only 39% of the enzymatic activity due to the significant loss of main chain interactions with S75 even though several of the side chain interactions have been retained with S75 (Fig. 2). The introduction of an Ile residue seems to have slightly increased flexibility in the vicinity of the mutated residue. On the other hand, in the V103I ALS variant structure, the side chain seems to have gained additional interactions with residues I46, I56 and F76 making the hydrophobic core more stable (Fig. 2). This provides some structural explanation for the altered catalytic activity for this variant.

An important structural feature of ANG is that a part of the active site (i.e., the C-terminal region of the molecule) is obstructed by residue Q117 (part of the pyrimidine recognition site)^6^. It has been postulated that movement of Q117 and adjacent residues is required for substrate binding and catalysis^42^. Mutational studies on C-terminal segment of ANG have also shown that shorter side-chain residues or deletion of residues affects the catalytic activity^47^ and previous structural studies have revealed that the segment containing residues 117–123 in the wild-type is highly mobile^48^. In the 3D structure of native ANG, R121 forms part of a peripheral sub-site for binding polynucleotide substrates at the C-terminal region’s closed conformation. The ALS associated variant R121C has significantly higher RNase activity (131%) as compared with wild-type ANG. In the crystal structure of the variant (Fig. 2), the smaller side-chain of cysteine appears to provide more flexibility for the C-terminal end of the molecule by destabilizing the closed conformation thus providing better access to substrates which would explain the enhanced RNase activity. Similar observations have been reported previously for the R121H ALS implicated variant^8^.

In summary, we have elucidated altered molecular interactions that underpin the abrogated catalytic efficiency or an increase in RNase activity (directly linked to ANG function) of eleven new variants of ANG implicated in PD and ALS using high resolution X-ray diffraction data. In our previous studies^6^,^16^,^17^ we have shown that the survival of the motor neurons and neurite extension are linked to the weak RNase activity. Abrogation of the RNase activity or an increase in RNase activity both have phenotypic consequences on the motor neurons leading to fragmented neurites in ALS settings. It is quite likely that similar effects could be anticipated in the case of PD setting which need further experimental study using ‘*in vitro*’ model systems. Meanwhile the observations made from the present study will provide the structural basis in understanding the biological roles for these variants which have been implicated in PD and ALS.

## Methods

### Expression and purification of native and ANG-PD/ALS variants

Wild-type and all ANG PD/ALS variants were prepared as reported in Holloway *et al*.^49^. Briefly, a synthetic gene for wild-type ANG with *E. coli* codon bias was inserted into pET-22b(+)(Novagen) for expression of the Met-(−1) form of the protein. The QuikChange site-directed mutagenesis method was used to introduce mutations. After DNA sequencing (Cogenics, UK; MWG, Germany) to confirm the presence of the mutations, mutant plasmids were used to transform *E. coli* BL21(DE3) cells. Bacterial cells were grown in Terrific Broth at 37 °C to an OD_600_ of 0.5–0.6, after which expression was induced by addition of isopropyl β-D-1-thiogalactopyranoside (IPTG) to a final concentration of 1 mM and incubation was continued for ~3 hours before harvesting. The target proteins from inclusion bodies were extracted, refolded and purified by SP-Sepharose chromatography followed by C4 reversed-phase HPLC^49^. Purified proteins were lyophilized and dissolved in HPLC grade water. All ANG variants behaved similarly to the wild-type protein during purification. Concentrations of all recombinant proteins were determined from UV absorbance, using an ε280 value of 12,500 M^−1^cm^−1^ calculated by the method described by Pace *et al*.^50^ All purified protein masses were experimentally confirmed using the electrospray ionisation mass spectrometry (ESIMS). In the case of R121C variant, the mass spectrometry data showed that an additional glutathione residue was attached to the free cysteine residue.

### Ribonucleolytic activity assay

Activity toward tRNA was determined by measuring the formation of acid soluble fragments as described by Shapiro *et al*.^5^ Assay mixtures contained 2 mg ml^–1^ yeast tRNA (Sigma), 0.1 mg ml^–1^ bovine serum albumin (BSA) and 0.05, 0.1, 0.2, 0.3, 0.4, or 0.5 μM test protein in 33 mM Na-Hepes, 33 mM NaCl, pH 7.0. After 2 h of incubation at 37 °C, reactions were terminated by the addition of 2.3 vol ice-cold 3.4% perchloric acid, the mixtures were centrifuged at 13000 g for 10 min at 4 °C, and the absorbance of the supernatants were measured at 260 nm (Table 4). Each data point used in the calculation were the mean of 3 measurements. In all cases, the standard deviation is less than 2% of the mean. The activities listed are the means of the values calculated for 0.3, 0.4 and 0.5 μM variant.

### X-ray crystallographic studies

Variant ANG proteins were crystallised using the sitting drop method, some conditions were optimised with hanging drop (for detailed conditions used see Table 2). Diffraction data were collected at 100 K, with poly(ethylene glycol) 4000 (30% w/v) or 25% glycerol as a cryoprotectant, on beamlines i02, i03, i04, i04–1 and i24 at Diamond Light Source (Oxon, UK) equipped with ADCS Quantum 315 and Dectris PILATUS detectors (Dectris, Switzerland). CCD data and some PAD datasets were indexed and integrated with Xia2^51^,^52^ pipeline 3dii using XDS^53^ while the remaining PAD data were indexed and integrated with DIALS^54^. Higher multiplicity X-ray diffraction data were collected for more recent datasets to maximise the resolution of the data. Some datasets have higher values for R_merge_ or statistics derived from R_merge_ than might traditionally be accepted due to poorer quality diffraction or higher multiplicity, however, as resolution cut-offs based on data quality were selected to achieve a minimum CC_1/2_ of 0.4^55^,^56^, these statistics were deemed acceptable. Resolution cut-offs were also selected based on a minimum outer shell completeness of approximately 70%. All data were scaled with AIMLESS^56^,^57^. Space group assignments were confirmed with ZANUDA^58^.

Initial phases were obtained by molecular replacement with PHASER^59^ using 1ANG as the starting model^6^. Further refinement and model building were carried out with REFMAC5^60^ and COOT^61^, respectively. Visible bound water and ligand molecules (from the crystallisation medium) were modelled into the electron density using difference density map (Fo-Fc) after all the protein atoms were built. With each data set, a set of reflections (between 5–10%) was kept aside for the calculation of R_free_. All the structures were validated using MolProbity validation tool^62^. There were no residues in the disallowed region of the Ramachandran plot except <1% in the H13R, Q77P, F100I and H114R variant structures. Crystallographic data statistics are summarized in Table 2. All figures were rendered and RMSDs between 4AOH, the high resolution native ANG structure, and variant structures were calculated using PyMol (DeLano Scientific LLC, San Carlos, CA, USA). Solvent accessibilities were calculated with Areaimol^63^.

## Additional Information

**Accession codes:** The atomic coordinates and structure factor amplitudes have been deposited with the Protein Data Bank (www.pdb.org) (PDB ID codes for ANG variants are- 5M9A, 5M9C, 5M9G, 5M9J, 5M9M, 5M9P, 5M9Q, 5M9R, 5M9S, 5M9T and 5M9V for H13R, K40R, K54R, K60E, Q77P, T80S, R95Q, F100I, V103I, H114R and R121C respectively).

**How to cite this article:** Bradshaw, W. J. *et al*. Structural insights into human angiogenin variants implicated in Parkinson’s disease and Amyotrophic Lateral Sclerosis. *Sci. Rep.* **7**, 41996; doi: 10.1038/srep41996 (2017).

**Publisher's note:** Springer Nature remains neutral with regard to jurisdictional claims in published maps and institutional affiliations.

## Figures and Tables

**Table 1:** Reported ANG mutations implicated in PD and ALS.

Human ANG PD/ALS variant	Country of origin	Reference
Signal peptide		
***M(−24)I***	***Italian***	***27,28***
F(−13)L	German	25
F(−13)S	Italian	27,28
**V(−12)A**	**European, North American**	21
G(−10)D	Dutch	21
**G(−8)D**	**European, North American**	21
***P(−4)S***	***North American***	***27,29***
P(−4)Q	Belgian	21
**A(−1)P**	**North American**	30
Mature protein		
*Q12L*	Irish, Scottish	18
H13R*	**European, North American**	21
*K17E*	Irish, Swedish	18
***K17I***	***Irish, Scottish, Northern American, Dutch, German, Belgian***	***18,21,23,30***
**D22V**	**European, North American**	21
*S28N*	Northern American	29
*R31K*	Irish, English	18
C39W	European	18
*K40I*	Irish, Scottish	18,19
K40R*	European, North American	21
*I46V*	Scottish, Italian, French, German, Swedish	18,21,25, 26, 27, 28
*K54E*	German	25
K54R*	**European, North American**	21
K60E*	**North American**	30
Q77P*	**North American**	30
T80S*	Dutch	21
R95Q*	**European, North American**	21
F100I*	Dutch	21
*P112L*	Northern American	29
V103I*	Chinese	20
*V113I*	Italian	27
H114R*	Italian	27
R121C*	***Italian***	***21,22***
*R121H*	French	26

Colour codes: ANG-ALS variants, **ANG-PD variants** (bold), ***ANG- implicated in both ALS and PD variants*** (bold-italic).

*Crystal structures of ANG-ALS variants presented in this report.

*In italics*- Crystal structures of ANG-ALS variants presented in a previous report by Thiyagarajan *et al*.^8^.

**Table 2:** Crystallographic data for ANG PD/ALS variants.

Variant	H13R	K40R	K54R	K60E	Q77P	
Crystallisation conditions	0.2 M Na/K tartrate 20% PEG 3350	0.4 M Na/K tartrate 0.1 M Na citrate, pH 5.5 20% PEG 4000	0.2 M Na/K tartrate 0.1 M HEPES, pH 7.0 3% Propanol 20% PEG 8000	0.2 M Na/K tartrate 0.1 M Na cacodylate, pH 6.5 25% PEG 4000	0.2 M ammonium sulphate 0.1 M Bis-tris, pH 5.5 25% PEG 3350	
Space group	P2_1_2_1_2	C222_1_	C222_1_	C222_1_	P1	
Cell dimensions (a, b, c in Å, α, β, γ, in °)	37.4, 85.8, 32.9, 90, 90, 90	82.8, 116.1, 37.4, 90, 90, 90	82.9, 118.8, 37.1, 90, 90, 90	83.1, 117.1, 37.3, 90, 90, 90	50.2, 50.3, 62.2, 103.4, 103.6, 110.4	
No. molecules in ASU	1	1	1	1	4	
Resolution range (Å)	42.8–1.94	58.0–2.05	32.5–2.28	58.5–1.90	56.8–1.65	
*R_merge_	0.193 (1.938)	0.066 (0.359)	0.127 (0.846)	0.059 (0.542)	0.263 (1.760)	
R_meas_	0.244 (2.526)	0.077 (0.496)	0.152 (1.019)	0.075 (0.679)	0.281 (1.933)	
R_pim_	0.149 (1.604)	0.038 (0.340)	0.058 (0.400)	0.034 (0.314)	0.099 (0.777)	
CC_1/2_	0.986 (0.466)	0.998 (0.887)	0.994 (0.904)	0.992 (0.626)	0.988 (0.622)	
Mean < I/σI>	4.9 (1.2)	15.0 (2.5)	6.7 (1.1)	14.2 (2.6)	7.3 (1.9)	
Completeness	99.2 (91.2)	95.4 (69.6)	100.0 (100.0)	96.2 (71.2)	96.6 (70.4)	
Total number of reflections	34867 (1963)	71536 (1702)	51806 (5108)	67772 (3096)	898984 (25028)	
Unique reflections	8160 (493)	11150 (616)	8730 (842)	14137 (684)	60548 (2326)	
Multiplicity	4.3 (4.0)	6.4 (2.8)	5.9 (6.1)	4.8 (4.5)	14.8 (10.8)	
**Refinement statistics**						
R_work_/R_free_	23.2/29.7	18.5/23.0	22.0/27.1	17.8/21.6	23.2/27.1	
Average B-factor (Å^2^)						
Protein	30.9	36.6	48.2	30.9	22.3	
Solvent	33.8	44.7	42.1	39.5	28.8	
Ligand	51.3	54.1	65.2	35.9	52.7	
Number of atoms						
Protein	955	1022	998	1034	3941	
Solvent	30	72	32	99	330	
Ligand	10	17	17	10	107	
RMSD						
bond length (Å)	0.010	0.010	0.011	0.010	0.012	
bond angle (°)	1.41	1.42	1.52	1.31	1.50	
Ramachandran statistics (%)						
Favoured	96.5	95.4	96.4	98.1	93.9	
Allowed	2.6	4.6	3.6	1.9	5.7	
Outliers	0.9	0	0	0	0.5	
**Ligands	TAR	TAR, PEG	TAR, PEG	TLA	SO4, PGE, EDO, BTB	
**PDB code**	**5M9A**	**5M9C**	**5M9G**	**5M9J**	**5M9M**	
**Variant**	**T80S**	**R95Q**	**F100I**	**V103I**	**H114R**	**R121C**
Crystallisation conditions	0.2 M ammonium sulphate 0.1 M Bis-tris propane, pH 5.5 25% PEG 3350	0.2 M ammonium sulphate 0.1 M Na cacodylate, pH 6.5 30% PEG 8000	0.2 M Na/K tartrate 0.1 M Na cacodylate, pH 6.5 20% PEG 4000	0.2 M Na/K tartrate 0.1 M MES, pH 6.5 20% PEG 4000	0.01 M Na borate, pH 8.51.1 M Na citrate	0.2 M Na/K tartrate 0.1 M Bis-tris propane, pH 6.5 16% PEG 4000
Space group	P2_1_2_1_2	P2_1_2_1_2	P1	C222_1_	P3_2_21	C222_1_
Cell dimensions (a, b, c in Å, α, β, γ, in °)	82.4, 37.3, 42.8, 90, 90, 90	82.6, 37.4, 43.1, 90, 90, 90	30.9, 34.8, 52.9, 89.7, 84.2, 84.3	82.8, 115.9, 37.3, 90, 90, 90	81.4, 81.4, 86.0, 90, 90, 120	82.5, 119.0, 37.4, 90, 90, 90
No. molecules in ASU	1	1	2	1	2	1
Resolution range (Å)	42.8–1.80	82.6–1.35	52.7–1.44	67.4–1.85	70.5–2.20	67.8–1.70
*R_merge_	0.135 (0.528)	0.105 (1.394)	0.096 (0.171)	0.199 (0.994)	0.110 (0.712)	0.097 (1.752)
R_meas_	0.156 (0.662)	0.144 (1.613)	0.136 (0.242)	0.226 (1.324)	0.121 (0.913)	0.120 (2.190)
R_pim_	0.057 (0.342)	0.031 (0.595)	0.096 (0.171)	0.076 (0.723)	0.036 (0.436)	0.070 (1.300)
CC_1/2_	0.984 (0.650)	0.998 (0.434)	0.989 (0.937)	0.993 (0.461)	0.994 (0.681)	0.997 (0.470)
Mean < I/σI>	9.0 (2.0)	11.1 (1.5)	6.5 (3.9)	6.3 (1.0)	12.1 (1.8)	7.3 (0.9)
Completeness	95.7 (77.9)	96.6 (71.6)	95.1 (80.1)	90.7 (76.3)	95.8 (75.9)	99.7 (98.6)
Total number of reflections	77958 (1844)	348247 (7220)	91164 (3685)	91131 (1991)	156743 (3932)	106361 (5445)
Unique reflections	12236 (596)	29013 (1033)	37557 (1635)	14125 (723)	16435 (1101)	20751 (1063)
Multiplicity	6.4 (3.1)	12.0 (7.0)	2.4 (2.3)	6.5 (2.8)	9.5 (3.6)	5.1 (5.1)
**Refinement statistics**						
R_work_/R_free_	23.9/28.5	15.1/17.9	17.2/19.5	20.8/26.5	18.2/23.6	20.9/23.9
Average B-factor (Å^2^)						
Protein	21.3	18.4	12.1	33.1	49.9	31.7
Solvent	28.5	33.1	23.4	39.1	47.7	41.0
Ligand	54.9	40.7	14.3	54.0	66.5	36.4
Number of atoms						
Protein	1020	1071	1989	1010	1986	1026
Solvent	73	154	236	67	67	110
Ligand	20	51	47	10	11	10
RMSD						
bond length (Å)	0.011	0.013	0.011	0.012	0.011	0.011
bond angle (°)	1.46	1.75	1.62	1.49	1.60	1.37
Ramachandran statistics (%)						
Favoured	98.1	97.1	95.6	97.3	96.2	96.3
Allowed	1.9	2.9	3.9	2.7	3.4	3.7
Outliers	0	0	0.4	0	0.4	0
**Ligands	SO4	SO4, PEG, PGE	TAR, TLA, PEG	TAR	GOL, BO4	TLA, CYS
**PDB code**	**5M9P**	**5M9Q**	**5M9R**	**5M9S**	**5M9T**	**5M9V**

*Values in parentheses refer to the highest resolution shell.

**Ligand definition based on observation in the electron density maps from each variant structure: TAR, D-tartrate; PEG, di-ethylene glycol; TLA, L-tartrate; SO4, sulphate ion; PGE, tri-ethylene glycol; EDO, ethylene glycol; BTB, bis-tris methane; GOL, glycerol; BO4, borate ion; CYS, cysteine (glutathione linked).

**Table 3:** Hydrogen bond and van der Waals contact residues in ANG and ANG-PD and ANG-ALS variants.

Variant	Potential hydrogen bonding residues			Potential van der Waals contact residues		
	Retained	Gained	Lost	Retained	Gained	Lost
H13R	F9, I46	N43, Q117	T44	F9, L10, T11, K40, T44, F45, I46, L115, Q117		
K40R	L35, P38, Y94	Q12	I42, N43	M30, P38, I42, N43, Y94	Q12, L35	H13
K54R	K50, I56, C57	Y6		R51, S52, I56, C57, L111, P112	Y6, K50	
K60E		E58		A55, G62, K73	E58	
Q77P	H47		R21	I46, H47, G99, F100		R21
T80S	T44, T97			C26, N43, R95, A96, T97, F120		I42, T44, K82
R95Q	K82			D23, C81, K82, T97		H84
F100I						S75, F76, Q77
V103I	S72			R70, I71, V78, V105, I119, F120	I46, I56, F76	
H114R	A106			F9, V105, A106	L69	D116
R121C				S118, I119, F120		

^*^Note- The electron density for R121C is not optimal while R122 shows poor density and P123 is not visible in the electron density map (i.e., disordered) in the R121C variant structure. Also, the cysteine residue with a glutathione molecule bound to the free cysteine was observed in the variant structure.

**Table 4:** Structural deviation and solvent accessibility changes caused by ANG PD and ALS variants.

	ANG ALS/PD variant	(% RNase activity)	Solvent accessibility area of corresponding residue in ANG PD/ALS variant (Å^2^)	Solvent accessibility area of corresponding residue in Native ANG (Å^2^)	Root-mean-square deviation against native ANG^**^ (Å)
1	H13R	0.0	22.7	9.7	0.27
2	K40R	6.3	79.9	56.8	0.19
3	K54R	48.5	85.7	81.1	0.26
4	K60E	79.6	135.0	153.9	0.17
5	Q77P	60.1	39.0	78.2	0.49
6	T80S	78.7	8.5	0.0	0.23
7	R95Q	52.0	123.5	131.6	0.18
8	F100I	39.1	109.7	104.5	0.31
9	V103I	54.1	0.0	0.1	0.25
10	H114R	1.6	127.0	87.0	0.44
11	R121C	131.2	58.1	140.9	0.20

^*^Percentage RNase activity to yeast tRNA in comparison to the native ANG Met^−1^ ANG (100%).

^**^Against PDB-4AOH native ANG structure by Thiyagarajan *et al*.^8^.
